# Private Sector Engagement for Tuberculosis Services in Latin America: A Systematic Review

**DOI:** 10.3390/ijerph22050681

**Published:** 2025-04-25

**Authors:** Carlos Podalirio Borges de Almeida, Leonid Lecca, Courtney M. Yuen

**Affiliations:** 1 Faculty of Public Health, Institute of Health and Biological Sciences, Federal University of Sul e Sudeste do Pará, Marabá 68500-000, Brazil; 2 Department of Global Health and Social Medicine, Harvard Medical School, Boston, MA 02115, USA; llecca_ses@pih.org (L.L.); courtney_yuen@hms.harvard.edu (C.M.Y.); 3 Socios En Salud Sucursal Peru, Lima 15001, Peru; 4 Division of Global Health Equity, Brigham and Women’s Hospital, Boston, MA 02115, USA

**Keywords:** tuberculosis, Latin America, private sector, public–private sector partnerships

## Abstract

**Objectives:** Private sector engagement has been shown to improve tuberculosis diagnosis and treatment outcomes in Asia and Africa, but systematic reviews in 2015 and 2017 identified only two reports from Latin America. We conducted a systematic review to identify descriptions of private sector engagement interventions for tuberculosis in Latin America. **Methods:** We systematically searched for reports on private sector engagement for tuberculosis services in Spanish- and Portuguese-speaking countries of the Western Hemisphere. On 1 November 2024, we searched PubMed, Embase, LILACS, and SciELO, with terms related to tuberculosis, the private sector, and eligible countries. We double-reviewed abstracts and full-text articles and classified private sector engagement mechanisms according to an established framework. **Results:** We identified seven documents describing five distinct interventions for private sector engagement in 10 countries. The most common engagement mechanism was technical support to increase awareness, knowledge, or capacity in the private sector. Intervention goals included promoting collaboration, ensuring adherence to national guidelines, increasing referrals to the public sector, and reducing tuberculosis drug sales in private pharmacies. Three impact evaluations found evidence of improved referral to the public sector. **Conclusions:** We found few reports of private sector engagement interventions for tuberculosis in Latin America, suggesting missed opportunities for collaborations to expand and improve tuberculosis service delivery. A lack of impact assessments suggests a dearth of evidence on the best models for private sector engagement to advance tuberculosis elimination in the Latin American region.

## 1. Introduction

A substantial amount of health care is delivered in the private sector in all regions of the world other than Europe [1]. Use of private sector services is common even among poorer segments of the population, even though out-of-pocket payments are higher in the private sector compared to the public sector [2]. The achievement of universal health coverage in almost all Latin American countries has relied on a public–private mix of both financing and service provision, with models varying across countries [3].

Government national tuberculosis programs (NTPs) oversee surveillance and tuberculosis (TB) service delivery in the public sector for all countries. National population-based surveys from Asia and Africa have shown that 30–85% of people with TB seek care first in the private sector [2]. Small-scale studies from Latin America have similarly found that 20–45% of people with TB seek care first in the private sector, although the evidence is sparser [4,5,6,7,8]. In some countries—particularly in Asia—the private sector provides nearly half of all first-line treatment, while other countries prohibit the sale of TB medications in the private sector [2]. Engagement of the private sector by NTPs is necessary to facilitate prompt diagnosis and appropriate treatment by reaching people affected by TB where they prefer to seek care. Since 2001, the World Health Organization has recommended that NTPs engage private providers in TB service delivery, supporting NTPs to do so by producing toolkits for engaging private providers [9].

Private sector engagement interventions in Asia and Africa have been shown to improve TB diagnosis and treatment outcomes [10]. In contrast, evidence on the impact of private sector engagement interventions to improve TB service delivery is not well described in Latin America. Systematic reviews from 2015 and 2017 [10,11] identified only two reports from Latin America [12,13], with all others coming from Asia and Africa. Latin American countries represent a distinct epidemiologic and health system context from the Asian and African countries in which the vast majority of research around private sector engagement for TB has occurred. While the majority of countries in Africa and Asia are classified as having high TB incidence (≥100 per 100,000 population), the majority of Latin American countries are classified as having a moderate TB incidence (10–100 cases per 100,000 population annually) and are thus closer to the pre-elimination phase of the epidemic [14]. Understanding the existing evidence on private sector engagement in Latin America is important for advancing TB elimination in the region. We therefore performed a systematic review to describe the evidence around effective models for private sector engagement for TB in Latin America.

## 2. Methods

We focused our review on Latin America because of the dearth of evidence from Latin America in previous systematic reviews. Our rationale for a new review focused on this region was that the searches for the prior reviews [10,11] were conducted 10 years earlier than ours, and that they included only documents in English or Chinese, which would exclude literature published in regional Spanish- and Portuguese-language journals. We followed the Preferred Reporting Items for Systematic reviews and Meta-Analyses (PRISMA) guidelines for a scoping review (Table S1). The scoping review methodology was more appropriate than the systematic review and meta-analysis methodology, as the objective was to conduct a systematic literature review to describe models for private sector engagement in Latin America rather than assessing the effectiveness of a specific approach defined *a priori* [15]. We did not register the protocol.

We defined private sector engagement interventions as programs that actively facilitate collaboration between a government TB program and the private sector, or that seek to improve delivery of services for the diagnosis, treatment, or prevention of TB in the private sector. We considered the private sector to include private health facilities and medical professionals, pharmacies and drug-sellers, and any other entities described as being part of the private sector in individual documents. While different definitions of Latin America exist, we focused on Spanish- or Portuguese-speaking countries in the Western Hemisphere (Costa Rica, El Salvador, Guatemala, Honduras, Mexico, Nicaragua, Panama, Argentina, Bolivia, Chile, Colombia, Ecuador, Paraguay, Peru, Uruguay, Venezuela, Cuba, Dominican Republic, and Brazil). Our rationale was that health systems in formerly colonized countries have structural characteristics derived from their colonial history, so experiences with private sector engagement from Africa and Asia are least applicable to countries formerly colonized by Spain or Portugal.

We searched PubMed, Embase, LILACS, and SciELO on 1 November 2024, with topic and text terms related to TB, as well as “private sector” as a topic or “private” as text (Table 1). We placed no restriction on the publication date. Searches in PubMed and Embase also included terms to specify Latin America and its constituent countries. Searches in LILACS and SciELO translated all search terms in English, Spanish, and Portuguese. We manually searched the citations of relevant review articles to identify additional references.

**Figure 1:**
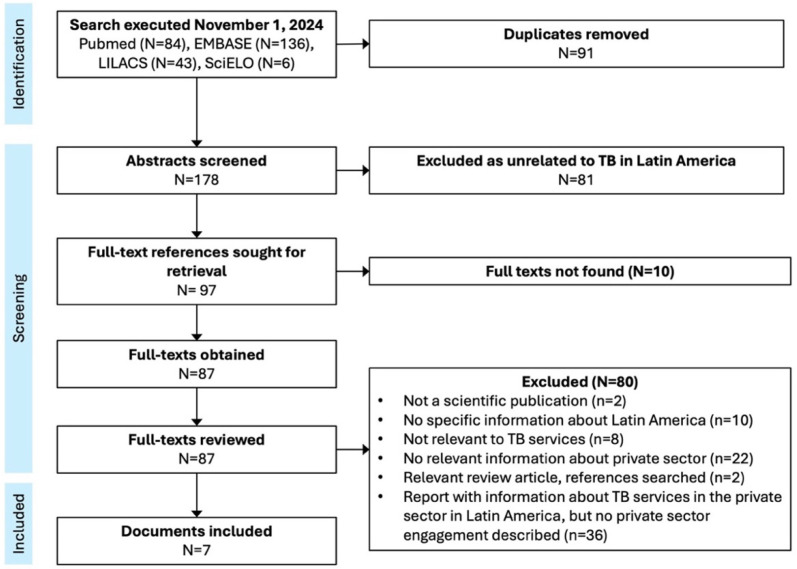
PRISMA diagram.

We reviewed abstracts to include references relevant to TB in Latin America, excluding studies not from Latin America (per our definition), studies specifically about a condition other than TB in humans, clinical case reports, and general epidemiologic or program profiles that only mentioned TB as one of many health conditions. We reviewed full-text references in any language. We included scientific publications describing an intervention promoting private sector engagement for TB services, applying a hierarchical list of exclusion criteria (Figure 1).

We extracted data on the setting, targets, activities, objectives, and impact of each intervention. We categorized engagement approaches according to the framework defined by the 2015 systematic review [10], including financial, material, or technical support; contracting (formal or informal) to define roles between public and private sectors; and formation of multi-sectoral stakeholder groups. We chose *a priori* to present a narrative synthesis without any meta-analysis given the anticipated heterogeneity of reports identified. CPBA and CMY conducted independent double review for abstracts and full texts, and independent double data extraction, with discrepancies resolved by consensus. We synthesized findings in a table summarizing approaches for private sector engagement classified according to the aforementioned framework, indicating whether any impact analysis was conducted.

## 3. Results

We identified 178 unique references through database searches and none through manual citation review (Figure 1). We reviewed 87 full texts and identified 7 reports describing private sector engagement for TB in 10 countries of Latin America (Table 2). These reported five unique interventions, two aimed at private medical doctors [16,17,18] and three at private pharmacies or drug sellers [12,13,19,20]. Interventions were implemented between 1998–2010.

None of the interventions involved financial support from the government to private sector entities. Four of the five interventions described technical support to increase awareness, knowledge, or capacity in the private sector. Two of the interventions described formal or informal contracting mechanisms in which private sector providers or facilities agreed to specific roles in collaborating with the NTP. Two of the interventions described establishment of a multi-sector stakeholder group.

The goals of the interventions varied. A training initiative in nine countries led by the International Union Against Tuberculosis and Lung Disease (IUATLD) and the Pan-American Health Organization (PAHO) in 1998–2002 sought to increase private physicians’ TB knowledge and encourage collaboration with NTPs [16,17]. Interventions aimed at pharmacies and drug-sellers in Bolivia, Peru, and the Dominican Republic sought to increase referral of clients with TB symptoms to public services for testing and treatment [12,13,19,20]; the intervention in Bolivia also sought to decrease sales of TB drugs in private pharmacies. An intervention in Colombia prioritized ensuring high-quality treatment in the private sector [18].

We found three formal impact evaluations of engagement interventions. Following a training course in El Salvador, private physicians reported increased suspicion of TB and increased referral to public services. Training interventions for pharmacists and drug sellers in the Dominican Republic and Bolivia resulted in improved referral of simulated patients. While case notifications increased in the year following the intervention in the Dominican Republic, the referral engagement initiative in Bolivia was discontinued because referrals from pharmacies comprised only 5% of TB notifications.

## 4. Discussion

We found relatively few reports of private sector engagement interventions for TB in Latin America, and scant evidence on the impact of engagement efforts. At a regional level, PAHO and IUATLD supported training of private providers during 1997–2002 to promote better collaboration NTPs [17,21], and PAHO has maintained guidance promoting private sector engagement. PAHO’s 2016 “Framework for tuberculosis control in large cities of Latin America and the Caribbean” recognized health sector fragmentation as a key barrier to TB service delivery and recommended that NTPs establish multisectoral stakeholder groups including the private sector, increase engagement with private sector providers, and offer quality control services to private laboratories [22]. While it is possible that these activities have taken place, our review found little scientific literature describing their implementation or evaluating their impact.

Recent studies from multiple Latin American countries have identified lack of coordination between public and private sector providers as a barrier to accessing TB services. Studies from Honduras and the Dominican Republic have documented confusion when patients received information or diagnostic procedures in private clinics that differed from what they received in the public sector [23,24], suggesting that training initiatives in the 1990s to ensure that private providers provided services consistent with national TB guidelines may not have been sustained in some countries [17]. Moreover, in countries like Peru where treatment of TB in the private sector is limited or nonexistent [25,26], a lack of formal referral mechanisms for people diagnosed with TB in the private sector can complicate the process of accessing treatment [27].

These studies suggest that better private sector engagement by NTPs in Latin America is needed to improve TB diagnosis and treatment. Establishing formal referral mechanisms from the private sector to public services for the testing and treatment of TB may increase TB diagnosis and treatment initiation outcomes [28,29]. Regulatory mechanisms that promote involvement of the private sector in TB care are also important. While NTPs understandably wish to maintain strict standards of care to ensure quality TB services, overly restrictive and burdensome requirements can discourage private sector providers from providing these services [25].

Private pharmacies are an important target for engagement, as self-medication of respiratory symptoms is common. Since 2005, efforts to engage private pharmacies to increase TB diagnoses have become institutionalized across many countries of Asia and Africa, with engagement strategies incorporated into national strategic plans following the success of pilot interventions [11]. While we identified a few reports of interventions to engage pharmacists in Bolivia, Peru, and the Dominican Republic, it is not clear if these efforts are currently widespread or institutionalized. If not, NTPs should incorporate guidelines to clearly outline the identification and referral processes for TB, specifically targeting pharmacists in the private sector and private pharmacy associations. Furthermore, it is crucial to educate pharmacy professionals to recognize the symptoms of TB and facilitate the referral of affected individuals to public TB services, and they can also be engaged to support TB treatment [29].

Understanding the policy landscape is important for advancing private sector engagement in Latin America. While we excluded policy documents from our review because policy documents do not capture the reality of practice, two policy documents that our search identified described an annual training activity plan under a public–private alliance in El Salvador [30] and a collaborative mechanism for treating TB in the private sector in Peru [31]. To better understand the implications of these and other policies will require a policy review that assesses current NTP mandates and collaborative mechanisms, situates those mechanisms within the heterogeneous models of incorporating private sector care within different countries’ health systems, and ascertains the extent to which mandated collaborative activities are implemented.

Our review is subject to limitations. Our search may not have captured all local phrases used to refer to the private sector, which might specifically have reduced our ability to find articles published in national or local journals. We also encountered a relatively high proportion of non-retrievable documents, although the majority of these were published before 1990 and hence are potentially less relevant to the present. We recognize that our search yielded relatively few references despite reasonably broad search terms. However, the fact our search identified the two articles identified by previous systematic reviews as well as others excluded from those reviews suggests the validity of our search strategy and points to a real scarcity of literature on the topic. The studies included in our review described short-term interventions, most did not conduct impact evaluations, and none examined impact on long-term outcomes, such as TB incidence or mortality rates. Going forward, more robust longitudinal studies of the impact of sustained interventions will be important for building the evidence base on private sector engagement for TB in this region.

## 5. Conclusions

There are missed opportunities for private sector engagement around TB service delivery in Latin America. It is important to advocate for mechanisms that foster collaboration between public and private sectors in the realm of TB care, such as the establishment of referral systems to public services for the testing and treatment of TB and policies that promote rather than restrict collaboration. While establishing new mechanisms requires resources, many Latin American countries are potentially well positioned to leverage existing financing structures that incorporate the private sector in social health insurance and universal health coverage programs [3]—a strategy currently being explored in Asian countries as a means to transition private sector engagement programs away from reliance on international donor funding [32]. Furthermore, conducting studies that evaluate the impact of public–private sector engagement interventions in Latin America will be essential for understanding what engagement strategies are effective in the Latin American context for improving TB outcomes as more Latin American countries enter the pre-elimination phase of the TB epidemic. Lastly, it is important to encourage the dissemination of both successful and unsuccessful private sector engagement models for TB in Latin America through scholarly publications. Some experiences may exist that have not been documented in the scientific literature, yet possess significant potential to enhance our understanding of this issue.

## Figures and Tables

**Table 1:** **Search strategy in PubMed and Embase**. As LILACS and SciELO are regional databases, searches included only blocks 1 and 2, with translation of all phrases into Spanish and Portuguese.

	PubMed	Embase
1	Tuberculosis [MeSH Terms] or tuberculosis [Tiab] or TB [Tiab]	‘tuberculosis’/exp or ‘tuberculosis’: ab, ti or ‘TB’: ab, ti
2	Private sector [MeSH Terms] or private [Tiab]	‘private sector’/exp or ‘private’: ab, ti
3	Latin America [MeSH Terms] or Latin America [Tiab] or any constituent country as either MeSH term or title/abstract phrase	‘South and Central America’/exp or any constituent country in the title or abstract
4	1 and 2 and 3	1 and 2 and 3

**Table 2:** Characteristics of documents describing private sector engagement for TB service delivery in Latin America.

Country, Document Type, Intervention Year [Reference]	Engagement Target and Lead Implementor	Engagement Mechanism and Duration	Desired Outcome	Impact Evaluation
El Salvador, article, 1998–1999 [16]	**Target**: Private health facilities or providers **Lead implementor**: IUATLD (France)	**Technical support**: Training workshop for private physicians **Contracting**: Agreement to collaborate with NTP (unclear if formal or informal) **Duration**: 3-day workshop	Increase TB knowledge, promote adherence to NTP guidelines	Self-reported increased suspicion of TB, increased referral to NTP, following NTP guidelines, and notifying cases
Multi-country *, article, 1998–2002 [17]	**Target**: Private health facilities or providers **Lead implementor**: IUATLD (France)	**Technical support**: Training workshop for private physicians **Contracting**: Signed memorandum of understanding **Duration**: 3-day workshop	Increase TB knowledge and encourage collaboration with NTP	No formal evaluation, but NTPs expressed opinion that private providers are engaged and following guidelines
Bolivia, two articles **, 2001–2002 [12,19]	**Target**: Pharmacies or drug sellers **Lead implementor**: Institute for Tropical Medicine (Belgium)	**Technical support**: Presentation on TB given during pharmacists’ meeting **Multi-stakeholder group**: Discussion among researchers, NTP, department of health, and members of pharmacists’ association resulted in association’s recommendation to stop TB drug sales in pharmacies and refer clients to public services. **Other**: Introduction of referral slips **Duration**: Stakeholder meetings over 6 months to reach consensus on stopping TB drug sales, referral slip intervention for 2 months	Decrease TB drug availability in private pharmacies, improve referrals to NTP	Significant decrease in pharmacies selling TB drugs, significant increase in referral of simulated patients, low contribution of pharmacy referrals to total case notifications
Colombia, article, 2008 [18]	**Target**: Private health facilities or providers **Lead implementors**: Secretariat of Public Health and CIDEIM (Colombia)	**Multi-stakeholder group**: Establishment of an advisory committee for treatment of drug-resistant TB in both public and private sectors **Other**: Drug resistance survey conducted that included private sector patients **Duration:** Not described	Improve detection and management of drug-resistant TB	None
Dominican Republic, article, 2010 [13]	**Target**: Pharmacies or drug sellers **Lead implementor**: KNCV Tuberculosis Foundation (Netherlands)	**Material support:** Posters, brochures, and other materials with TB messages provided **Technical support**: Educational workshops and outreach visits on recognizing TB symptoms and effectiveness and availability of free TB treatment **Contracting**: Voluntary pledge signed **Other**: Introduction of referral slips **Duration:** 6 months	Increase TB knowledge, improve referrals to NTP	Significant increase in composite score when tested with simulated patients, increase in case notification rate post-intervention
Peru, conference abstract, year not reported [20]	**Target**: Pharmacies or drug sellers **Lead implementors**: Cayetano Heredia and Pedro Ruiz Gallo Universities (Peru)	**Technical support**: Educational SMS messages **Duration:** Not described	Increase TB knowledge	None

* Mexico, El Salvador, Honduras, Guatemala, Nicaragua, Peru, Dominican Republic, Costa Rica, and Bolivia; the El Salvador experience is described in reference [15]. ** Two articles published on the same intervention. Implementor abbreviations: IUATLD = International Union Against Tuberculosis and Lung Disease, CIDEIM = Centro Internacional de Entrenamiento e Investigaciones Médicas.

## Supplementary Materials

The following supporting information can be downloaded at: https://www.mdpi.com/article/10.3390/ijerph22050681/s1. Table S1: PRISMA-ScR checklist [33].
